# Supplementary figures and images for: Differentially expressed microRNAs during the differentiation of muscle-derived stem cells into insulin-producing cells, a promoting role of microRNA-708-5p/STK4 axis
Source: PLoS One. 2022 Apr 8;17(4):e0266609. doi: 10.1371/journal.pone.0266609 (PMC8992996; doi:10.1371/journal.pone.0266609)

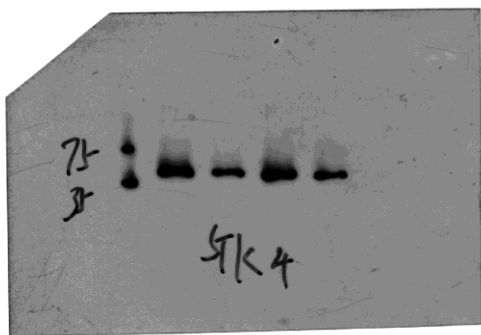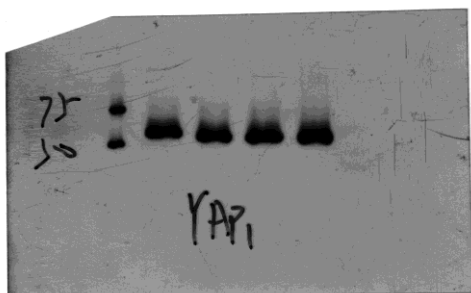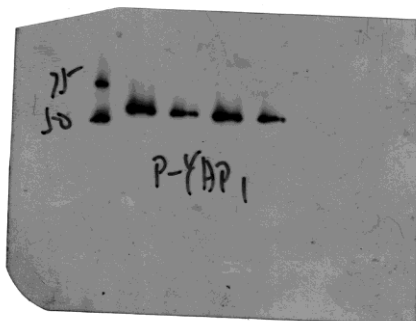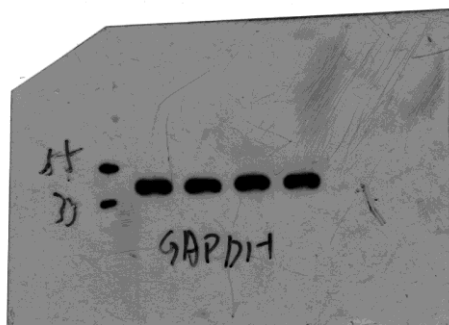

Supplement: S1 Raw images — (PDF) [file pone.0266609.s001.pdf]
