# Supplementary material for: Differentially expressed microRNAs during the differentiation of muscle-derived stem cells into insulin-producing cells, a promoting role of microRNA-708-5p/STK4 axis
Source: PLoS One. 2022 Apr 8;17(4):e0266609. doi: 10.1371/journal.pone.0266609 (PMC8992996; doi:10.1371/journal.pone.0266609)
Supplement: Attachment — Submitted filename: Response to Reviewers.docx [file pone.0266609.s003.docx]

**Response to reviewers**

Thanks for giving us the opportunity to submit a revised draft of the manuscript “Differentially expressed microRNAs during the differentiation of muscle-derived stem cells into insulin-producing cells, a promoting role of microRNA-708-5p/STK4 axis” for publication in the Journal of PLOS ONE. We appreciate the time and effort that editors and reviewers dedicated to providing feedback on our manuscript and are grateful for the insightful comments on and valuable improvements to our paper. We have incorporated most of the suggestions made by the reviewers. Those revisions are highlighted in the manuscript with tracked changes. Please see below, in blue, for a point-by-point response to the reviewers’ comments and concerns.

Journal Requirements:

Please review your reference list to ensure that it is complete and correct. If you have cited papers that have been retracted, please include the rationale for doing so in the manuscript text, or remove these references and replace them with relevant current references. Any changes to the reference list should be mentioned in the rebuttal letter that accompanies your revised manuscript. If you need to cite a retracted article, indicate the article’s retracted status in the References list and also include a citation and full reference for the retraction notice.

**R:** We have carefully reviewed the reference cited in this manuscript, and ensure that it is complete and correct.

Additional Staff Editor Comments (if provided): PLOS ONE does not provide copyediting or proofs of accepted manuscripts. We therefore recommend that you carefully review your manuscript and correct any language errors at this time.

**R:** We have carefully reviewed this manuscript, and corrected language errors.

Reviewer #1: There is still one comment which need to be addressed.

Statistical Analysis:

Line 182: Each experiment was performed at least three independent repetitions.

This sentence reflects that Authors were confirmed about number of replicates. Why "at least"? Why not that "All the experiments were performed with 3 replicates"?

Pls revise the sentence.

**R:** Thanks for pointing this out. We have revised the sentence “Each experiment was performed at least three independent repetitions.” to “All the experiments were performed with three independent repetitions.” in line 181.

Reviewer #2: The authors have answered all the comments satisfactorily. Now, it can be considered for publication.

**R:** Authors appreciate reviewer’s valuable comments and approval for publication.
